# Stakeholder Perceptions of Point-of-Care Ultrasound Implementation in Resource-Limited Settings

**DOI:** 10.3390/diagnostics9040153

**Published:** 2019-10-18

**Authors:** Anna M. Maw, Brittany Galvin, Ricardo Henri, Michael Yao, Bruno Exame, Michelle Fleshner, Meredith P. Fort, Megan A. Morris

**Affiliations:** 1 Division of Hospital Medicine, University of Colorado, Aurora, CO 80045, USA; 2 Alma Mater Hospital, Gros Morne 4210, Haiti; galvibr@gmail.com (B.G.); ricardsh93@yahoo.fr (R.H.); brunoexame@gmail.com (B.E.); 3 Division of Engineering and Applied Science, California Institute of Technology, Pasadena, CA 91125, USA; michael.yao@atriaconnect.org; 4 Division of General Internal Medicine, University of Pittsburgh Medical Center, Pittsburgh, PA 15260, USA; fleshnerm@upmc.edu; 5 Department of Health Systems, Management and Policy, Centers for American Indian and Alaska Native Health, Colorado School of Public Health, Aurora, CO 80045, USA; meredith.fort@cuanschutz.edu; 6 Adult and Child Consortium for Health Outcomes Research and Delivery Science, University of Colorado School of Medicine, Aurora, CO 80045, USA; megan.a.morris@cuanschutz.edu

**Keywords:** point-of-care-ultrasound, ultrasound, implementation

## Abstract

Background: Nearly half of the world lacks access to diagnostic imaging. Point of care ultrasound (POCUS) is a versatile and relatively affordable imaging modality that offers promise as a means of bridging the radiology gap and improving care in low resource settings. Methods: We performed semi-structured interviews of key stakeholders at two diverse hospitals where POCUS implementation programs had recently been conducted: one in a rural private hospital in Haiti and the other in a public referral hospital in Malawi. Questions regarding the clinical utility of POCUS, as well as barriers and facilitators of its implementation, were asked of study participants. Using the Framework Method, analysis of interview transcripts was guided by the WHO ASSURED criteria for point of care diagnostics. Results: Fifteen stakeholders with diverse roles in POCUS implementation were interviewed. Interviewees from both sites considered POCUS a valuable diagnostic tool that improved clinical decisions. They perceived barriers to adequate training as one of the most important remaining barriers to POCUS implementation. Conclusions: In spite of the increasing affordability and portability of ultrasounds devices, there are still important barriers to the implementation of POCUS in resource-limited settings.

## 1. Introduction

It has been estimated that approximately half of the world lacks access to even basic diagnostic imaging [1]. Among the factors responsible for what is termed the “radiology gap” is the high cost required to procure and maintain most radiology equipment and the specialized expertise of necessary personnel such as radiologists and technicians [2].

Studies show that 80–90% of imaging needs can be addressed with X-ray and ultrasound alone [3,4]. Ultrasound is particularly versatile and is the least expensive of all imaging modalities [2]. Common applications of ultrasound that routinely change management decisions in low resource settings [5,6] include the diagnosis of pneumonia, extrapulmonary tuberculosis, parasitic infections, rheumatic heart disease, and ectopic pregnancy [7,8,9]. In addition, as noncommunicable diseases such as heart disease become more prevalent in low- and middle-income countries, so too does the utility of ultrasound in these settings [10].

Recognizing ultrasound as the most promising modality to bridge the radiology gap, the World Health Organization (WHO) published a series of documents beginning in the 1990s designed to facilitate the implementation of ultrasound more broadly [11,12]. At that time, the WHO warned that operator training was one of the biggest barriers to appropriate implementation.

Over the last 20 years, ultrasound has become even more portable and much less costly, allowing for the development of a new practice called point-of-care ultrasound (POCUS) [13], in which images are acquired and interpreted by the treating clinician at the bedside. POCUS is a growing field in Europe and the United States, with emerging evidence that it improves time to diagnosis and decreases cost in high-resource settings [14,15]. In contrast to consultative ultrasound, which is technician-performed and interpreted by a radiologist, POCUS answers focused diagnostic questions using easily performed techniques that can be quickly interpreted at the bedside. Because POCUS addresses focused questions and is not a comprehensive evaluation of an organ system, image acquisition and interpretation are easier to master than traditional consultative ultrasound. This aspect of POCUS reduces one of the remaining significant barriers to ultrasound’s widespread use in resource-limited settings: extensive training.

The reduced price of hand-held ultrasound machines, which cost approximately 10,000 USD in contrast to standard ultrasound machines which are about 50,000 USD [16], makes POCUS a particularly good fit for resource-limited settings, as does its immediate availability of results and lack of reliance on additional infrastructure and personnel (e.g., technicians, radiologists, and laboratories). Continued improvements in cost, portability, and battery life all will all serve to reduce barriers to its implementation over time. Given the dynamic nature of factors that influence the implementation of POCUS and the expanding applications of its use, the purpose of this study is to determine the current barriers and facilitators of its implementation in low-resource settings. 

## 2. Materials and Methods

### 2.1. Study Sample and Setting

The Institutional Review Board at University of Colorado deemed this study not Human Subjects research. We obtained written consent from all participants prior to their interview. We interviewed key stakeholders from 2 diverse hospital settings in which POCUS implementation programs had recently been conducted. One site was in a private rural 60 bed hospital in Haiti and the other was an 800 bed referral hospital in Malawi. Key stakeholders from both sites were interviewed as part of the study. We defined a key stakeholder as an individual who in some way influences the implementation of POCUS. The following types of stakeholders were included in the study: program initiators (individuals who established the POCUS implementation program), POCUS instructors, hospital administrators, provider trainees, and local physician leaders. 

### 2.2. Data Collection

Data for this study were collected between July 2019 and September 2019. The WHO ASSURED criteria [17] (Table 1) were used to shape the interview guide, data coding and analysis. These criteria were developed by the WHO Sexually Transmitted Diseases Diagnostics Initiative to describe the characteristics of the perfect point-of-care diagnostic test in resource-limited settings. However, we feel it outlines important considerations for any type of diagnostic test and highlights many of the reasons why POCUS is uniquely positioned to be the most effective imaging modality for low-resource settings.

Semistructured interviews were conducted with key stakeholders to assess their perspective on point of care ultrasound implementation in their local setting and more broadly. Interviews were conducted by phone and in person. Some interviews were conducted in English and some in Creole, depending on the language spoken by the interviewee. Those conducted in English were conducted by AMM. Those conducted in Creole were performed by BG, who both conducted the interviews and translated answers into English during the interview. Interviews were conducted until thematic saturation was reached: when no additional themes were immerging from the interviews. All interviews were conducted using the same interview guide (see Supplementary Materials).

### 2.3. Data Entry and Analysis

The interviews were audio recorded and transcribed verbatim. We used a framework analysis [18] and a largely deductive approach. Our coding framework was informed by the WHO ASSURED criteria. We were also open to new themes that may have arisen inductively from the data. Our coding process was guided by consensus qualitative research methods [19]. The consensus research approach has the following features: data were collected through open-ended questions in semi-structured interviews, interviews were analyzed to achieve consensus of at least 2 analysts, an outside auditor (a qualitative expert not integrally involved in the study) supervised the process to help maximize the validity of the findings, thematic content analysis was performed by AMM, study site leaders BG (Haiti) and MF (Malawi) reviewed the categorization of themes obtained from stakeholders at their respective sites to ensure the validity of the analysis, and MAM acted as the qualitative expert supervising the analysis.

Patterns of POCUS implementation facilitators and barriers were identified from participants’ interviews. First, participants’ responses were coded into framework categories, which were then grouped into themes.

## 3. Results

A total of 15 interviews were conducted by key stakeholders (Table 2) at two sites located in a small private rural hospital in Haiti and a large public referral hospital in Malawi. Interview length ranged from 20 to 45 min.

The study aimed to understand factors that may affect the implementation and sustainability of POCUS in low-resource settings. Guided by the WHO ASSURED criteria for point-of-care diagnostics in resource limited settings, core themes were identified (Table 3).

### 3.1. Affordability

The costs associated with several aspects of implementation were considered barriers. Expenditures included costs related to adequate accessibility to machines and training.

In addition, interviewees in Haiti expressed the potential of cost-savings to both the patients and hospital associated with ultrasound as it allowed for local diagnosis and treatment rather than referral to a larger facility.

#### 3.1.1. Cost Related to Machines

Stakeholders expressed that although machines were more accessible than they had been previously, this cost was still considered a barrier to implementation. This was the case in part because even if providers had received training in the past, if there was no machine in their current practice setting they would not be able to maintain their skill.

*“After the people who are training the doctors leave, we need to be sure that there are ultrasound machines available for everyone to practice. Even if there are ultrasounds at some hospitals, doctors often take different jobs and if those ultrasounds aren’t available at all hospitals then everything that the doctor learned they might forget because they don’t have the ultrasound available to them. Therefore, they would lose this skill.”*

#### 3.1.2. Cost Related to Training

The cost related to training was also perceived as a significant barrier to implementation.

*“It was the gynecologist we had on staff who said, ‘You know, I’ve always wanted to go to a more formal ultrasound program, but when I looked at them, they were like 1,500, 2,500 U.S. dollars.’ He goes, ‘I can’t afford that’.”*

*(Haiti)*

#### 3.1.3. Cost to the Patient

Interviewees associated with the Haiti site felt POCUS improved cost from the perspective of the patients because it allowed them to receive a diagnosis locally thereby avoiding both the expense of travel to a referral hospital and the cost of a consultative ultrasound once there.

*“Advantage was for patients because they used to have to travel and spend a lot of money to be able to have ultrasound diagnostics in other locations. Now that we have the diagnosis here, they don’t have to travel and they don’t have to pay a fee for the diagnostics.”*

*(Haiti)*

In contrast, in Malawi, because it was a public hospital, there was no additional cost to the patients because all care was free.

*“The hospital that I work at is a public hospital so everything is included. For things that are able to be done at the public hospital it’s not an issue…. for the purposes of the ultrasounds, and the X-rays, they’re not charged anything.”*

*(Malawi)*

#### 3.1.4. Revenue Generation for the Hospital

Interviewees in Haiti also noted that being able to offer ultrasound may generate revenue for the hospital because when they are able to receive a diagnosis locally, they can remain at the hospital for treatment.

*“If we could increase the utilization of the service in the hospital it would be a huge benefit because we would become more competent in ultrasound and we could take on more patients…. therefore, keeping more patients in the hospital. Not having to refer them. And also, the hospital would be able to make more money because we are not having to send people away.”*

*(Haiti)*

### 3.2. Accuracy (Sensitivity and Specificity)

All interviewees from both sites believed POCUS improved diagnosis and management decisions significantly.

*“Actually I think that the Point of Care Ultrasound is very, very important in taking care of the patient particularly in the resource limited settings. We don’t really have access to imaging modalities like MRI, CT. With ultrasound we can get a lot of information for most parts of the body. You can scan the lungs, you can scan the heart, you can scan the abdomen, you can scan pretty much everywhere. I think that this is actually a very helpful tool that would help improving healthcare in settings like this.”*

*(Haiti)*

*“We are looking at a lot of people being misdiagnosed. We kind of have a lot of deaths that may be would have been averted if we’d have really known what was happening.”*

*(Malawi)*

*“Most people here do paras and thoras completely blind. So training them to use POCUS to do procedures more safely would be another big benefit.”*

*(Malawi)*

### 3.3. User-Friendly

Once proficient, trainees felt POCUS exams were quick and easy to perform. However, all interviewees felt that adequate training was currently an important barrier to use. Subthemes included (1) language barriers between trainees and instructors; (2) limited time for trainees to attend trainings when offered because of clinical responsibilities; (3) lack of continuity of staff who have been trained. 

#### 3.3.1. Language

Interviewees in Haiti reported language as a barrier to training, in that the majority of instructors spoke English but almost all of the trainees spoke Creole and French. Language was not reported as a barrier by stakeholders from the hospital in Malawi where the instructors and trainees all spoke English.

*“One challenge was for the foreigners who came to teach the lesson. We don’t speak the same language, so there was a language difficulty.”*

*(Haiti)*

#### 3.3.2. Time to Attend Trainings

Interviewees at both sites reported that clinical responsibilities were an important barrier to attending training sessions when offered.

*“The biggest challenge was that we were trying to take care of the patients at the same time as learning because we still had a role at the hospital. We didn’t have time off.”*

*(Haiti)*

*“In general, our medical department right now is super short-staffed and so the interns are really overworked. So the time it takes to train is another big barrier.”*

*(Malawi)*

#### 3.3.3. Lack of Clinician Continuity

Interviewees at both sites reported lack of clinician continuity was an important barrier to implementation and sustainability.

*“So it’s a little bit harder because if we were to train them, then they’re gone in three months. So they’re not a sustainable part of the process.”*

*(Malawi)*

*“They come and go now and then, and this is actually another challenge. Some of the doctors trained have already left the hospital.”*

*(Haiti)*

### 3.4. Rapid and Robust

Interviewees expressed that one of the important benefits of POCUS is that results are immediately available. This was an important advantage at the rural hospital in Haiti, where there was no consultative ultrasound available and patients were often unable to return for test results. It was important at the referral hospital in Malawi as well because there were such long waits for consultative ultrasound performed by radiology.

*“The advantages are tremendous because so often our X-ray machine isn’t working, and/or it’s a terrible X-ray machine, the images aren’t clear. Plus we’re so limited in referral, if you’re in the States, if you have a patient who is injured, even if they’re not at one hospital, you can quickly transfer them to another hospital. In Haiti it’s not so much the case. In addition to the cost of care can be astronomical. So free point of care testing that can be rapid, on the spot is a huge advantage for diagnosis and treatment.”*

*(Haiti)*

*“If maybe most of the clinicians, can be trained on how to use an ultrasound, it’ll be far better, because instead of sending the patient to radiology they can be doing it on their own. Because most of the time it will be like, ‘Okay, it’ll be done like in the next three days, because there a lot of patients that are waiting.’” *

*(Malawi)*

### 3.5. Equipment-Free

Interviewees expressed that the hand-held ultrasounds were easier to maintain than other forms of radiology (X-ray, commuted tomography, and consultative ultrasound). Interestingly, interviewees related times that POCUS findings were useful as surrogates for laboratory results because basic labs were not reliably available at either site. Due to its small size, loss of the hand-held ultrasound was more of a concern than need for repair or maintenance. However, although neither site reported an instance in which a hand-held ultrasound had required maintenance or repair, interviewees expressed concern that there was no local technician to repair it should the need arise.

*“I think one of the biggest challenges with respect to the ultrasound and with respect to a lot of other things is that equipment tends to walk off. And so I think in terms of buy-in, there needs to be somebody who’s kind of the gatekeeper and keeps the device from disappearing.”*

*(Malawi)*

*“And if there’s a problem with the equipment, who’s qualified to repair it or to troubleshoot? Because we don’t have technicians of that nature available in Haiti.”*

*(Haiti)*

### 3.6. Delivered

Interviewees at both sites reported that POCUS was extremely portable, all but eliminating time and resources spent on delivery and installation which are important barriers to implementation with other radiologic modalities [2]. Stakeholders at the Haiti site reported having a hand-held device available allowed for diagnosis locally instead of needing to refer patients to a larger hospital with consultative ultrasound or other imaging modalities. Stakeholders at the site reported being able to obtain imaging on patients too sick to come to the hospital via home visits and those in the hospital but too sick to travel to radiology.

*“For my other job… they do home visits. So there have been a couple times that there are people who can’t come to clinic because of whatever, because they’ve had a stroke or et cetera. There’ve been a couple times that I’ve gone out with residents and taken the ultrasound with me and that is really, really useful. So I mean, I think that there would be a real benefit to that.”*

*(Malawi)*

*“Now that it’s in the hospital, they don’t even have to leave the hospital. Therefore, they’re not having to spend this money and have more money to buy the medicine that they need. Basically, they can get all the care that they need in one place inside of the hospital.”*

*(Haiti)*

### 3.7. Inductive Themes

#### 3.7.1. Development and Support of Local Experts

One inductive theme that emerged from stakeholders in both Haiti and Malawi was a recommendation to support a few local providers to undergo significant training in POCUS and then offer them support to train other local providers.

*“So would there have been a possibility of appointing one physician and maybe giving them an extra stipend to encourage them, or sign a contract saying ‘Hey, we’re going to train you in this. This is your three year contract and these are the objectives of hiring you as this. Not only are you going to see patients, but you’re going to be the lead for this and you’re responsible to train other physicians as they come in.’ So there is, even if we have turnover, there is a plan to engage new physicians as we bring them aboard.”*

*(Haiti)*

*“Getting the Registrars to be excited about POCUS because they’re the one who will really be doing it. They’re the ones that teach the interns, they’re the ones who teach the med students. Getting a few of them trained and then going from there would be the best strategy. … The barrier is that they get paid virtually nothing so they all end up basically taking second jobs at private hospitals or private clinics and then end up not being at work as much. … I think if we were able to provide one or two people with extra money to do POCUS training and that was their incentive, I think that would be huge.”*

*(Malawi)*

#### 3.7.2. Remote Learning Technology

Another inductive theme that emerged was the potential but current limitations of the internet as a means of providing remote learning opportunities and addressing the barrier of training without local experts available.

*“We did develop this way of uploading all of our images to Google Drive and then creating a log and a spreadsheet of logging all of our images. Basically 250 images QA’d by people from the states. So, the goal ultimately would be to do that with local providers and I think it would work but there’s a few big barriers. The biggest of which is paying. We had to use data, straight phone data for it and it probably cost like $100 over the course of two months. Which isn’t a lot here but it’s not a sustainable thing for them to do. So that’s kind of a challenge.”*

*(Malawi)*

*“Actually it is a challenge, in such limited settings, even the Internet, we had the devices, they had an integrated software called ‘React’ that allows remote education, but we couldn’t use it properly because of the Internet. We had Internet access at the hospital, but it was a very low speed. We couldn’t really use it. Internet access as well is a barrier. Like with Internet we could have been able to do remote education, it would have been much easier to do it.”*

*(Haiti)*

## 4. Discussion

To our knowledge, this is the first study to explore the barriers and facilitators of POCUS implementation in resource-limited settings using qualitative methods. Our findings demonstrate the perceived clinical utility of POCUS, as well as the remaining barriers to implementation and sustainability. All stakeholders expressed the belief that POCUS had the potential to greatly improve management decisions and patient care but felt that the cost and time required for training were major barriers to implementation. Other barriers identified by study participants included the lack of continuity of providers at each site that would allow retention of an institutional expert to provide longitudinal mentorship as well as curriculum development and implementation which would be required for trainees to attain competency. Additional barriers included equipment maintenance and accessibility. This finding is consistent with other studies on this topic [20]. Interviewees also reported the local internet services were currently too slow, unreliable, or expensive to use effectively for remote learning. However, it is important to note that given the pace of advances in both remote learning technology and artificial intelligence applications [21], the former allowing for accelerated training and the later offering automated image interpretation, a swift decline in training as a barrier to POCUS implementation is expected.

Stakeholders at both sites recommended focusing resources on the training of a small number of local providers that could then become educators for their region, which would address many of the barriers expressed. This approach would provide: (1) longitudinal mentoring to other local providers; (2) someone who would be accountable for maintaining equipment; (3) elimination of language barriers between instructor and learner; and (4) reduce the cost related to providing training (e.g., resources that would otherwise be invested in travel and time of nonlocal experts may instead be invested into local champions).

### Limitations

There are several important limitations to our study. The first is that we only interviewed stakeholders associated with two POCUS implementation program sites. This may limit the generalizability of our results. However, the sites were diverse clinical environments, one a small rural private hospital and the other a large public referral hospital, located in different countries. Additionally, the barriers that emerged as themes included the cost of the machines, language barriers, and lack of continuity of experts which would be expected to apply to many low-resource settings, suggesting many of our findings are to some extent generalizable. The study also would have been improved by gaining a broader healthcare system perspective by interviewing people within the Ministry of Health in each of the countries represented to determine whether they consider POCUS implementation worth investing in. Additionally, because a third of the study participants and most of research team is from the United States it is possible that we failed to capture all relevant themes accurately due to cultural bias. Finally, some of our interviews were done by phone as opposed to in-person which may have resulted in loss of contextual or nonverbal information. However, given the subject matter, which is of a relatively concrete and unsensitive nature, we feel it is unlikely this greatly influenced the themes that emerged.

## 5. Conclusions

POCUS is considered a highly effective diagnostic tool that improves patient care by all stakeholders interviewed. Although more affordable, the cost of machines is still a barrier to implementation of POCUS, though the expense and time required for optimal training of local providers seems to be the most important current barrier. Our findings highlight the remaining barriers to implementation as well as offer potential strategies to overcome them.

## Figures and Tables

**Table 1:** WHO ASSURED Criteria.

Affordable (Affordable to the Communities Who Need It)
Sensitive
Specific
User-Friendly (Simple to perform requiring minimal training)
Rapid and Robust (Quick results that enable treatment on the first visit)
Equipment-free (minimal reliance on technological infrastructure)
Delivered (Ability to be delivered to those who need it; portable)

**Table 2:** Stakeholder interviewees and their role in POCUS implementation.

Type of Stakeholder	Description of Stakeholders
2 Program Founders	Both individuals were from the United States: 1 began the implementation program in Haiti and the other initiated the program in Malawi.
3 Instructors	All 3 were physicians from the United States with expertise in POCUS: 1 internist who taught in Haiti and 2 medical residents who taught in Malawi.
8 Trainees	All were local providers: 1 nurse practitioner, 2 residents, 1 internist, and 1 gynecologist who worked in Haiti, and 2 registrars and 1 intern who worked in Malawi.
2 Hospital Administrators	Both were associated with the site in Haiti.

**Table 3:** Themes identified from interview data.

Framework Domain	Subthemes	Subtheme by Site
Affordability	Cost and Cost Saving	
	cost of machines	*Haiti and Malawi*
		it was much easier to obtain machines by external funders than in the past
	cost of training	*Haiti and Malawi*
		local providers were unable to pay the cost of classes
	cost saving to patient	*Haiti*
		availability of POCUS allowed patients to be diagnosed locally instead of having to travel to the referral hospital
		*Malawi*
		POCUS exams did not affect cost to the patient as all care was free
	cost savings to hospital	*Haiti*
		availability of POCUS allowed patients who would normally be referred to another hospital, to be diagnosed locally which in turn allowed them to purchase care and medications locally
		*Malawi*
		POCUS did not affect cost or cost savings to the hospital
Accuracy	greatly improved diagnosis and management	*Haiti and Malawi*
		POCUS improved accuracy for many diagnoses at both hospitals, expediating appropriate treatment and making procedures safer
User-friendly	language barrier	*Haiti*
		language barrier between instructors and local providers was a barrier to training
		*Malawi*
		language was not a barrier in Malawi as both instructors and local providers spoke English
	requires significant training	*Haiti and Malawi*
		the amount of time required for training was considered an important barrier at both sites
	lack of continuity of staff	*Haiti and Malawi* lack of clinician continuity at both sites created a barrier to training as there was no expert consistently available to teach others
Rapid and Robust	greatly decreased time to imaging	*Haiti*
		no consultative ultrasound was available, therefore POCUS improved time to diagnosis as because patients did not have to travel to a referral hospital for an ultrasound
		*Malawi*
		POCUS allowed for more rapid diagnosis as there was a long wait for X-rays and consultative ultrasound
Equipment-free	no expertise to fix hand-held ultrasound locally	*Haiti and Malawi*
		concerns were expressed regarding what to do if a machine broke because there is no local expert to fix a hand-held ultrasound
	loss of hand-held ultrasound.	*Haiti and Malawi*
		concerns were expressed about the likelihood of a hand-held ultrasound being lost given its small size
Delivered	diagnosis in rural locations.	*Haiti*
		POCUS allowed patients to be diagnosed in a rural hospital setting without requiring travel to another larger hospital
	diagnosis of patients too ill to travel	*Malawi*
		POCUS allowed patients that could not physically be moved to radiology department to be diagnosed at referral hospital.
		POCUS also allowed diagnosis via home visits when patients were not able to travel to the hospital
Inductive Themes	development of local experts	*Haiti and Malawi*
		study participants recommended focusing resources on the development of local clinicians so that they can assume the role of local experts and can train other local clinicians
	remote learning technology	*Haiti and Malawi*
		the expense and unreliability of the internet limited access to remote learning

## Supplementary Materials

The following are available online at https://www.mdpi.com/2075-4418/9/4/153/s1: Interview Guide.
