# Supplementary material for: Stakeholder Perceptions of Point-of-Care Ultrasound Implementation in Resource-Limited Settings
Source: Diagnostics (Basel). 2019 Oct 18;9(4):153. doi: 10.3390/diagnostics9040153 (PMC6963438; doi:10.3390/diagnostics9040153)
Supplement: Supplementary file 1 [file diagnostics-09-00153-s001.pdf]

Stakeholder Interview Guide

1. Please tell me your experience with point of care ultrasound, POCUS, implementation?
2. Who are the important stakeholders in POCUS implementation?
3. What is your role in POCUS implementation?
4. What are the advantages of POCUS implementation?
5. What are the disadvantages of POCUS implementation?
6. What were the challenges of POCUS implementation?
7. Were these challenges overcome? If so how?
8. What are the advantages of POCUS to different stakeholders from your perspective (e.g. patients, providers, administrators, hospital, community)? Are they significant?
9. How do you feel about scaling up POCUS use in your hospital?
10. What are the anticipated advantages to wide spread implementation of POCUS in this country?
11. What factors do you think will determine whether POCUS is widely taken up by clinicians in this country?
12. What are the anticipated challenges to widespread implementation of POCUS in this country?
13. How can they be overcome?

What are the anticipated challenges of wide spread implementation of POCUS in Haiti?
